# Impaired WNT3/IGF‐1 Signaling in Dorsal Dentate Gyrus Contributes to Chronic Pain‐Related Cognitive Impairment

**DOI:** 10.1002/cns.70714

**Published:** 2025-12-18

**Authors:** Yajie An, Ying Wu, Xiangyong Li, Taihe Zhou, Heming Liu, Yao Xiao, Wenyu Lai, Yuxin Qiu, Xuhong Wei

**Affiliations:** ^1^ Department of Human Anatomy and Physiology, Zhongshan School of Medicine Sun Yat‐Sen University Guangzhou China; ^2^ Pain Research Center, Zhongshan School of Medicine Sun Yat‐Sen University Guangzhou China; ^3^ Infectious Department The Third Affiliated Hospital of Sun Yat‐Sen University Guangzhou China; ^4^ Department of Anesthesiology, the First Affiliated Hospital Sun Yat‐Sen University Guangzhou China; ^5^ Guangdong Provincial Key Laboratory of Brain Function and Disease, Zhongshan School of Medicine Sun Yat‐Sen University Guangzhou China

**Keywords:** astrocytes, cognitive impairment, dentate gyrus, IGF‐1, neurogenesis, neuropathic pain, PI3K/AKT signaling, WNT3

## Abstract

**Background:**

Neuropathic pain is frequently accompanied by cognitive deficits, but the neural circuits and molecular mechanisms linking nociceptive hypersensitivity to cognitive dysfunction remain incompletely understood. The hippocampal dentate gyrus (DG), a critical hub for adult neurogenesis and memory encoding, is emerging as a key substrate integrating pain processing and cognitive impairment, but how peripheral nerve injury disrupts DG homeostasis remains unclear.

**Methods:**

In this study, we used the spared nerve injury (SNI) mouse model to investigate the cellular and molecular mechanisms underlying comorbid neuropathic pain and cognitive deficits. Behavioral assessments, stereotaxic viral/drug delivery, Western blot, immunofluorescence staining, whole‐cell patch‐clamp recordings, ELISA, and primary astrocyte culture were employed to characterize phenotypic changes and regulatory pathways in the dorsal DG.

**Results:**

We found that SNI induced persistent mechanical hypersensitivity and cognitive impairment in mice, which was associated with reduced neurogenesis (decreased nestin^+^ radial glial‐like cells, DCX^+^ immature neurons, and NeuroD1 expression) and diminished intrinsic excitability of dorsal DG granule cells. Mechanistically, SNI triggered a “double hit” to dorsal DG homeostasis: (1) neuron‐specific insulin‐like growth factor‐1 (IGF‐1) resistance, characterized by increased serine phosphorylation of insulin receptor substrate‐1 (IRS1) at S612 in mature granule cells, reduced IGF‐1 levels, and impaired PI3K/AKT signaling; (2) downregulation of astrocyte‐derived WNT3, a key neurogenic regulator, which was mediated by proinflammatory cytokine TNF‐*α*. Therapeutically, local supplementation of IGF‐1 into the dorsal DG reversed SNI‐induced nociceptive and cognitive deficits via IGF‐1R/AKT‐dependent restoration of neurogenesis and granule cell excitability. Similarly, chemogenetic activation of dorsal DG astrocytes alleviated comorbid symptoms by enhancing WNT3 secretion, while chronic inhibition of these astrocytes mimicked SNI‐induced pain hypersensitivity, cognitive impairment, and disrupted neurogenesis. Exogenous administration of WNT3a recapitulated the therapeutic effects by activating AKT, independent of IGF‐1R signaling.

**Conclusion:**

Our findings identify that decreased WNT3 secretion from astrocytes in dorsal DG integrates nociceptive and cognitive dysfunction after nerve injury via crosstalking with the IGF‐1/AKT pathway. Targeting this WNT3/IGF‐1 axis may represent a promising therapeutic strategy for neuropathic pain and its cognitive sequelae.

## Graphical abstract

Schematic diagram depicting the mechanism of SNI‐induced comorbidity of pain and cognition decline. SNI increased TNFα release in the dorsal DG (1); TNFα trigger a “double hit” to dorsal DG homeostasis: On the one hand, TNFα induced IGF‐1 resistance in granule neuron. On the other hand, TNFα downregulated Wnt3 release in astrocytes in dorsal DG, which then decreased neurogenesis (2). This functional deficit likely contributes to both cognitive impairment (via disrupted memory circuit integration) and nociceptive sensitization through impaired descending pain modulation pathways.

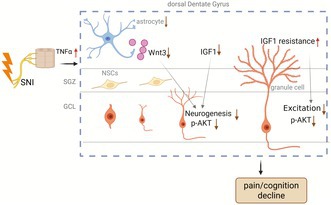

## 1 Introduction

Neuropathic pain, a chronic and debilitating condition arising from damage to the peripheral or central nervous system, affects millions worldwide and is frequently accompanied by cognitive comorbidities, including memory impairment, attention deficits, and executive dysfunction [1]. This dual pathology—persistent nociceptive hypersensitivity paired with cognitive decline—profoundly diminishes quality of life and remains challenging to treat, as existing therapies often fail to address both components simultaneously. A growing body of evidence points to the hippocampus, and specifically its dentate gyrus (DG) subregion, as a critical neural substrate linking pain processing and cognitive dysfunction [2, 3]. Structural neuroimaging studies in humans support this connection: patients with trigeminal neuralgia, a prototypical neuropathic pain condition, exhibit significant volume reductions in the DG compared to healthy controls [4], while preclinical models reveal that chronic pain disrupts DG neurogenesis [5, 6], impairs synaptic integrity [7], and reduces dopaminergic projections from the ventral tegmental area (VTA), collectively contributing to memory deficits [3].

The DG, a key locus for adult neurogenesis and memory encoding, relies on a delicate balance of cellular and molecular signals to maintain its functional integrity. Neural stem cells (NSCs) in the DG's subgranular zone (SGZ) generate new neurons throughout adulthood, a process critical for remodeling of neural circuits and closely related to cognitive function [8, 9]. This neurogenic cascade is tightly regulated by paracrine signals from glial cells, particularly astrocytes, and by trophic factors that support neuronal differentiation, maturation, and synaptic integration [10, 11]. Disruptions to these regulatory mechanisms—whether through inflammation, metabolic dysfunction, or trophic factor imbalance—can impair neurogenesis and disrupt DG circuit function, potentially driving both pain and cognitive pathology.

Among the molecular pathways governing DG homeostasis, insulin‐like growth factor‐1 (IGF‐1) signaling and WNT family proteins have emerged as key regulators. IGF‐1, a neurotrophic factor produced locally in the brain and systemically, acts via its receptor (IGF‐1R) to activate downstream PI3K/AKT signaling, promoting neuronal survival, differentiation, synaptic plasticity, and excitability [11, 12, 13]. Impaired IGF‐1 signaling, often characterized by aberrant serine phosphorylation of insulin receptor substrate‐1 (IRS1)—a critical adapter protein in the pathway—has been linked to cognitive decline in neurodegenerative diseases and metabolic disorders [14]. Similarly, astrocyte‐derived WNT3, a canonical WNT ligand, stimulates neurogenesis by upregulating early neuronal markers such as NeuroD1, and its downregulation in aging or disease states correlates with reduced DG neurogenesis [10, 15]. Notably, crosstalk between IGF‐1 and WNT signaling has been implicated in neurogenic regulation, with IRS proteins modulating WNT/*β*‐catenin activity and WNT ligands potentiating IGF‐1R activation [16, 17] suggesting that these pathways may function coordinately to maintain DG homeostasis.

Despite these advances, the mechanisms by which peripheral nerve injury disrupts IGF‐1 and WNT3 signaling in the DG—and how such disruptions contribute to comorbid pain and cognitive impairment—remain poorly understood. Chronic pain is known to trigger central neuroinflammation, with proinflammatory cytokines like TNF‐α altering astrocyte function and disrupting trophic factor secretion [18, 19, 20]. Additionally, TNF‐*α* and pain‐induced metabolic perturbations, such as elevated free fatty acids (FFAs) and sympathetic activation, can induce insulin/IGF‐1 resistance via serine phosphorylation of IRS1 [4, 21], potentially impairing neuronal responsiveness to trophic signals. However, whether these processes converge in the DG to create a “double hit” of reduced trophic support (via WNT3 loss) and impaired trophic responsiveness (via IGF‐1 resistance) remains untested.

In the present study, we hypothesized that peripheral nerve injury induces comorbid neuropathic pain and cognitive impairment by disrupting the WNT3/IGF‐1 axis in the dorsal DG. Using a mouse model of spared nerve injury (SNI), we characterized the cellular and molecular consequences of peripheral nerve injury on neurogenesis, granule cell excitability, and WNT3/IGF‐1 signaling in dorsal DG. We further tested whether targeted restoration of IGF‐1 or WNT3 function in the dorsal DG could reverse pain sensitization and cognitive deficits and explored the mechanistic interplay between these pathways. Our findings reveal a novel astrocyte–neuron signaling axis that integrates nociceptive and cognitive dysfunction, offering new therapeutic targets for neuropathic pain and its cognitive sequelae.

## 2 Materials and Methods

### 2.1 Animals

Eight‐week‐old male C57BL/6 mice (body weight 20–25 g) were obtained from the Animal Center of Sun Yat‐sen University (Guangzhou, China). The mice were group housed under specific pathogen‐free (SPF) conditions in a controlled environment (24°C ± 1°C, 50%–60% humidity) with a 12‐h light/dark cycle and provided *ad libitum* access to sterile water and standard laboratory chow. All animal protocols and experimental procedures were approved by the Institutional Animal Care and Use Committee (IACUC) of Sun Yat‐sen University (No. SYXK (yue) 2022–0081). All behavioral testing was performed during the light phase. The experimenters conducting the tests and analyses were blinded to the group assignments. Sample sizes were chosen in accordance with established standards for this experimental paradigm and are consistent with those commonly reported in prior literatures [22, 23].

This study was conducted using male mice only. We recognize the importance of considering sex as a biological variable, as mandated by funding agencies such as the NIH and journals including CNS Neuroscience & Therapeutics. Our decision to begin with male mice in this study was based on the following rationale. First, this study initially focused on the role of astrocyte‐derived WNT3 and IGF‐1 signaling in modulating neuronal plasticity during chronic pain‐induced cognitive impairment. However, existing literature indicates that astrocyte function, IGF‐1 signaling, and neuronal plasticity are each highly sensitive to fluctuations in ovarian hormones across the female estrous cycle [24, 25, 26]. For instance, estradiol upregulates key components of the IGF‐1 pathway, including IGF‐1R, Forkhead (FKHR), and Glut‐1 [27]. Furthermore, estrogen and progesterone regulate astrocytic morphology, gene expression, and metabolic activity [28]. The estrous cycle also modulates hippocampal processes fundamental to plasticity and cognition, such as spine dynamics, dendritic processing, and spatial coding. Therefore, including female subjects at this initial stage would have introduced a significant source of variability, potentially obscuring the core treatment effects we aimed to characterize. Second, this study serves as a foundational investigation to establish a clear phenotypic effect and mechanism of impaired WNT3/IGF‐1 signaling in dorsal dentate gyrus in chronic pain‐induced cognitive decline in a controlled setting. The insights gained here will provide an essential baseline for our planned future studies that will explicitly and rigorously examine sex differences in this model. Our approach is consistent with previously published studies in this specific field [15, 29, 30].

### 2.2 Spared Nerve Injury Model

Spared nerve injury was performed according to a previous study [31]. In brief, after anesthesia with isoflurane (1.5%–2.5%) in a mixture of 30% N_2_O and 70% O_2_, the sciatic nerve of the left hind limb was exposed at the trifurcation of peroneal, tibial, and sural branches. The common peroneal and tibial nerves were ligated and cut, whereas the sural nerve was left intact. For the animals in the sham group, the sciatic nerve was only exposed without ligation or cutting.

### 2.3 Behavioral Testing

Animals were acclimated to the experimental room and randomly assigned to experimental groups. All tests occurred between 9:00 a.m. and 5:00 p.m. A sequential battery of assessments—tactile sensitivity, dynamic mechanical allodynia, novel object recognition, and molecular analyses (immunofluorescence/western blot)—were performed on the same cohort of animals to minimize inter‐group variability.

### 2.4 Assessment of Tactile Sensitivity

Tactile sensitivity was assessed with the up‐down method. Mice were habituated to handling and the testing environment for ≥ 3 days prior to experiments. On the test day, after 45–60 min of acclimation, calibrated Von Frey filaments (0.04, 0.16, 0.4, 0.6, 1.0, 1.4, 2.0 g) were applied perpendicularly to the lateral plantar surface of the hind paw. Filaments were applied in either ascending or descending strengths to determine the filament strength closest to the hind paw withdrawal threshold. Each filament was applied for a maximum of 2 s at each trial. A positive response was defined as rapid paw withdrawal or licking. The tactile threshold for each hind paw was then calculated.

### 2.5 Dynamic Mechanical Test

Dynamic allodynia was quantified using a validated protocol [32]. Mice were habituated for 30 min in acrylic chambers before testing. Following 30‐min habituation in standardized von Frey testing chambers, the outside of the hind paw was gently stroked with a 5/0 brush, from heel to toe. The reaction was scored as follows: no response was counted as 0 point; a quickly moving or lifting the paw was counted as 1 point; continued lifting of the paw for more than 2 s or lifting the paw forcefully above the body was counted as 2 points; and flinching, licking, or flicking of the paw was counted as 3 points. Three trials per paw were conducted at 10‐min intervals, with the mean score representing the dynamic mechanical score.

### 2.6 Novel Object Recognition Test

The novel object recognition test was employed to assess short‐term memory ability. Briefly, the test was conducted in a black, round polyvinyl chloride box (diameter 80 cm, height 40 cm) with an open top. Animals were first habituated to the box for 10 min per day over two consecutive days. Subsequently, each animal was placed into the apparatus for the “sample phase,” where they were presented with two identical objects and allowed to explore freely for 5 min. The box and objects were thoroughly cleaned between trials to eliminate any residual olfactory cues. The time spent exploring each object was recorded, after which the animals were returned to their home cages. Following a 10‐min retention interval, one of the familiar objects was replaced with a novel object. The animal was then placed back into the box for the 5‐min “acquisition phase,” during which it could explore both the familiar and the novel object. The time spent exploring each object was measured again. A recognition index was calculated as the ratio of time spent exploring the novel object to the total time spent exploring both objects. An index above 0.5 indicated that the animal spent more time exploring the novel object, demonstrating recognition memory.

### 2.7 Cannula Implantation and Intracranial Injections

Mice were deeply anesthetized with intraperitoneal pentobarbital sodium (50 mg/kg) and placed in a stereotaxic frame (NARISHIGE, SR‐6R). After performing a midline scalp incision and craniotomy to access the skull, viral injections were targeted to the dorsal DG. Stereotaxic coordinates relative to bregma were as follows: anteroposterior −1.8 mm, mediolateral ±1.5 mm, dorsoventral −2.0 mm. Using a syringe pump, 100 nL of viral solution was delivered via a glass micropipette. For chemogenetic experiments, we used AAV2/8‐gfaABC1D‐hM3D(Gq)‐2A‐mCherry to activate astrocytes or AAV2/8‐gfaABC1D‐hM4D(Gi)‐2A‐mCherry to inhibit them (both from Obio Technology Corp.; titer: 1.34 × 10^13^ GC/ml).

Permanent guide cannulas (23gauge; RWD Life Science, Shenzhen, China) were implanted in dorsal DG for drug delivery, according to our previous studies [22, 33]. Drug infusions were administered using a Hamilton syringe connected to a 30‐gauge injector, delivering 0.5 μL over 1 min to lightly restrained mice. The injector remained in place for an additional minute post‐infusion to allow diffusion before the obturator was reinserted. Upon conclusion of the experiment, cannula placement was verified histologically; any mice with incorrect placement were excluded from the analysis.

### 2.8 Western Blotting

Following the defined survival time, mice were deeply anesthetized with 0.5% pentobarbital sodium (50 mg/kg). DG tissue was microdissected and homogenized on ice in 15 mM Tris buffer (pH 7.6) containing 250 mM sucrose, 1 mM magnesium chloride, 1 mM dithiothreitol, 2.5 mM EDTA, 1 mM EGTA, 50 mM sodium fluoride, 10 μg/mL leupeptin, 1.25 μg/mL pepstatin, 2.5 μg/mL aprotinin, 2 mM sodium pyrophosphate, 0.1 mM sodium orthovanadate, 0.5 mM phenylmethylsulfonyl fluoride, and a protease inhibitor mixture (Roche Molecular Biochemicals, Switzerland). The homogenates were sonicated and subsequently centrifuged at 13,000 × g for 15 min. Isolated proteins were separated by sodium dodecyl sulfate–polyacrylamide gel electrophoresis (SDS‐PAGE) and transferred onto a polyvinylidene fluoride (PVDF) membrane (Bio‐Rad Laboratories Inc., USA).

Blots were placed in blocking buffer [5% nonfat milk dissolved in Tris‐buffered saline (TBS) containing 50 mM Tris–HCl (pH 7.6) and 150 mM NaCl] for 1 h at room temperature and then incubated with primary antibodies targeting WNT3 (1:1000, 74,537, Santa Cruz, USA), Phospho‐AKT (Ser473, 4060, Cell Signaling technology), and AKT (4691, Cell Signaling technology) at 4°C. Membranes were washed with TBS‐Tween‐20 3 times and incubated with horseradish peroxidase‐conjugated secondary antibodies (rabbit anti‐goat, goat anti‐mouse, or goat anti‐rabbit IgG) for 2 h at room temperature.

Blots were developed with enhanced chemiluminescence (Clarity Western ECL Substrate, Bio‐Rad) and detected by a Tanon 5200 imager (Tanon Science & Technology Co. Ltd., Shanghai, China). Subsequent analysis was performed by Tanon MP software (Tanon Science & Technology Co. Ltd., Shanghai, China). Band intensities were quantified and normalized against a loading control (β‐actin) by an investigator blinded to the experimental conditions.

### 2.9 Astrocyte Culture

Astrocytes were cultured according to the following procedure. Under sterile conditions, the dorsal hippocampal region was dissected from mice. The tissue was minced into a paste‐like consistency and digested with 0.125% trypsin for 20 min. Using DMEM‐F12 medium as the culture medium, the cells were plated at a density of 1 × 10^6^ cells per milliliter onto 24‐well plates pre‐coated with poly‐L‐lysine. The plates were then placed in an incubator. The medium was replaced after 24 h. When cells reached 80% confluence, the astrocytes were subcultured for purification. The purified glial cells were seeded onto treated 24‐well plates and glass coverslips at a density of 1.0 × 10^5^ cells per square centimeter and cultured in the incubator for at least 10 days.

### 2.10 Immunohistochemistry

Upon completion of the survival period, mice were deeply anesthetized with 0.5% pentobarbital sodium (50 mg/kg). Transcardial perfusion was then performed through the ascending aorta, beginning with saline to flush the vasculature, followed by fixation with 4% paraformaldehyde in 0.1 M phosphate buffer. Cryoprotected brains were sectioned serially at 30 μm coronal planes on a cryostat (Leica CM 1900, Heidelberg, Germany). All of the cryostat sections were blocked with 2% donkey serum in 0.1% Triton X‐100 for 1 h at room temperature. The sections were then incubated with primary antibodies over two nights at 4°C. The following antibodies were used: p‐IRS (Ser616) (1:200, AB_2532503, Thermo Fisher), NeuroD1 (1:300, MA532626, Thermo Fisher), DCX (1:300, 18,723, Abcam), glial fibrillary acidic protein (1:500, 3670, Cell Signaling Technology), or neuronal specific nuclear protein (NeuN) (1:500, 177,487, Abcam). Afterwards, the double‐stained brain sections were incubated with secondary antibodies coupled to Alexa Fluor 488 or 594 (Invitrogen) for 1 h at room temperature and washed 3 times with PBS. For double‐immunofluorescence staining of p‐IRS (Ser616) with tissue markers, primary antibodies from the same batches as above were incubated together with antibody for NeuN, GFAP or goat polyclonal anti‐ionized calcium‐binding adaptor molecule 1 (Iba1, 1:1000, microglia marker, Abcam) for 2 nights at 4°C. After the incubation with primary antibodies, the sections were then treated with a mixture of Alexa Fluor 488 or 594 for 1 h at room temperature. The sections were finally protected by Antifade Mounting Medium with DAPI (Beyotime, China) under coverslips. Fluorescent images were obtained with a fluorescence microscope (Leica DFC350 FX camera). AAV‐transfected brain slices were only incubated with antibodies against mouse monoclonal GFAP (1:500).

The cultured astrocytes were fixed with 4% paraformaldehyde for 15 min and washed in PBS 3 times at room temperature. Then, the fixed cells were incubated with a mixture of WNT3 and GFAP antibody overnight at 4°C, followed by incubation with secondary antibodies. Images were captured with a Leica DFC350 FX camera. Four to six nonadjacent brain sections were randomly selected, and the optical density of positive IR was measured by Image J software (National Institutes of Health, Bethesda, MD), and the average was calculated.

### 2.11 Drugs

IGF‐1 (Peprotech, USA) was dissolved in 0.01 M PBS. WNT3a was purchased from MedChemExpress, USA. CNO purchased from APExBio, USA was dissolved in saline. JB‐1 and LY294002 (MCE, USA) were firstly dissolved in dimethyl sulfoxide (DMSO), stored as a stock solution of 0.1 M at—20°C, and diluted in sterile saline to the appropriate concentration immediately before administration. Recombinant murine TNFα was purchased from MCE, stored as a stock solution of 10 μg/mL at −70°C, and diluted to 100 ng/mL in 0.1% bovine serum albumin (BSA) in saline immediately before administration.

### 2.12 Whole‐Cell Recordings in Acute Brain Slices

Mice were anesthetized with isoflurane and transcardially perfused with an ice‐cold, carbogenated (95% O_2_/5% CO_2_) solution containing the following (in mM): 212.7 sucrose, 3 KCl, 1.25 NaH_2_PO_4_, 3 MgCl_2_, 1 CaCl_2_, 26 NaHCO_3_, and 10 dextrose. Coronal slices from the hippocampus (300 μm thick) were prepared from mice using a tissue slicer (Vibratome 3000; Vibratome) in ice‐cold dissection buffer, which was the same as the perfusion solution. The slices were then transferred to a holding chamber containing standard artificial cerebrospinal fluid (ACSF) at 35°C for a 30‐min recovery period. The standard ACSF contained (in mM): 124 NaCl, 3 KCl, 1.25 NaH₂PO₄, 1 MgCl₂, 2 CaCl₂, 26 NaHCO₃, and 10 dextrose.

Recordings were performed at 28°C–30°C using an Integrated Patch‐Clamp Amplifier controlled by Igor Pro 7 software (WaveMetrics), with signals filtered at 5 kHz and sampled at 20 kHz. Patch pipettes (2–4 MΩ) were filled with an internal solution composed of (in mM): 130 K‐gluconate, 10 KCl, 10 HEPES, 0.5 Na₃GTP, 4 MgATP, 10 Na‐phosphocreatine, and 0.2% biocytin (pH 7.2–7.4; 275–290 mOsm).

To measure excitability, current‐clamp recordings were targeted to a holding potential of −60 mV. Action potentials were evoked by injecting incremental current steps (20 pA increments) in the presence of synaptic blockers of CNQX (40 μM), APV (50 μM), bicuculline (10 μM), and strychnine (1 μM) in ACSF solution. Data were excluded when the resting membrane potential of neurons was less than −50 mV and action potentials did not have overshoot.

### 2.13 Enzyme‐Linked Immunosorbent Assay (ELISA)

The dorsal DG tissue was rapidly harvested and homogenized in phosphate‐buffered saline, followed by centrifugation at 4°C for 5 min at 5000 g. The collected supernatant from cultured astrocytes underwent centrifugation (1000 g, 15 min) to remove sediment. The supernatants after centrifugation were then used to measure the concentrations of IGF‐1 or WNT3 with the corresponding ELISA kits (IGF‐1: ab100695, Abcam, Cambridge, UK; WNT3: 48977 M2, Meimian, Jiangsu, China). The absorbance was read at 450 nm on a Wallac Victor 1420 multilabel counter (PerkinElmer Life Sciences, Norwalk, CT, USA). Sample concentrations were calculated by interpolating values from a concurrently run standard curve.

### 2.14 Quantification and Statistics

All the statistical analysis was performed using GraphPad Prism software version 8.0 (Prism 8.0; GraphPad, San Diego, CA). The sample sizes were determined based on our previous knowledge and experience with this design. Group sizes and experimental units are indicated in the result and figure legends. No outliers were observed. No data were missed or lost. Data are expressed as the mean ± standard error (SEM). The Shapiro–Wilk test was used to evaluate the data distribution, if *n* < 50. Otherwise, the Kolmogorov–Smirnov test was used. For normal distributions of variable values, two group comparisons were analyzed using unpaired *t*‐test and multiple group comparisons were analyzed using one–way analysis of variance (ANOVA), followed by Bonferroni post hoc correction. For nonparametric distributions, Mann–Whitney test was used for the two group comparisons, and the Friedman nonparametric one‐way ANOVA (with the post hoc Dunn test) was used for multiple comparisons. For all tests, *p* < 0.05 was considered significant.

## 3 Results

### 3.1 SNI Induces Comorbid Nociceptive Sensitization and Cognitive Dysfunction

**FIGURE 1:**
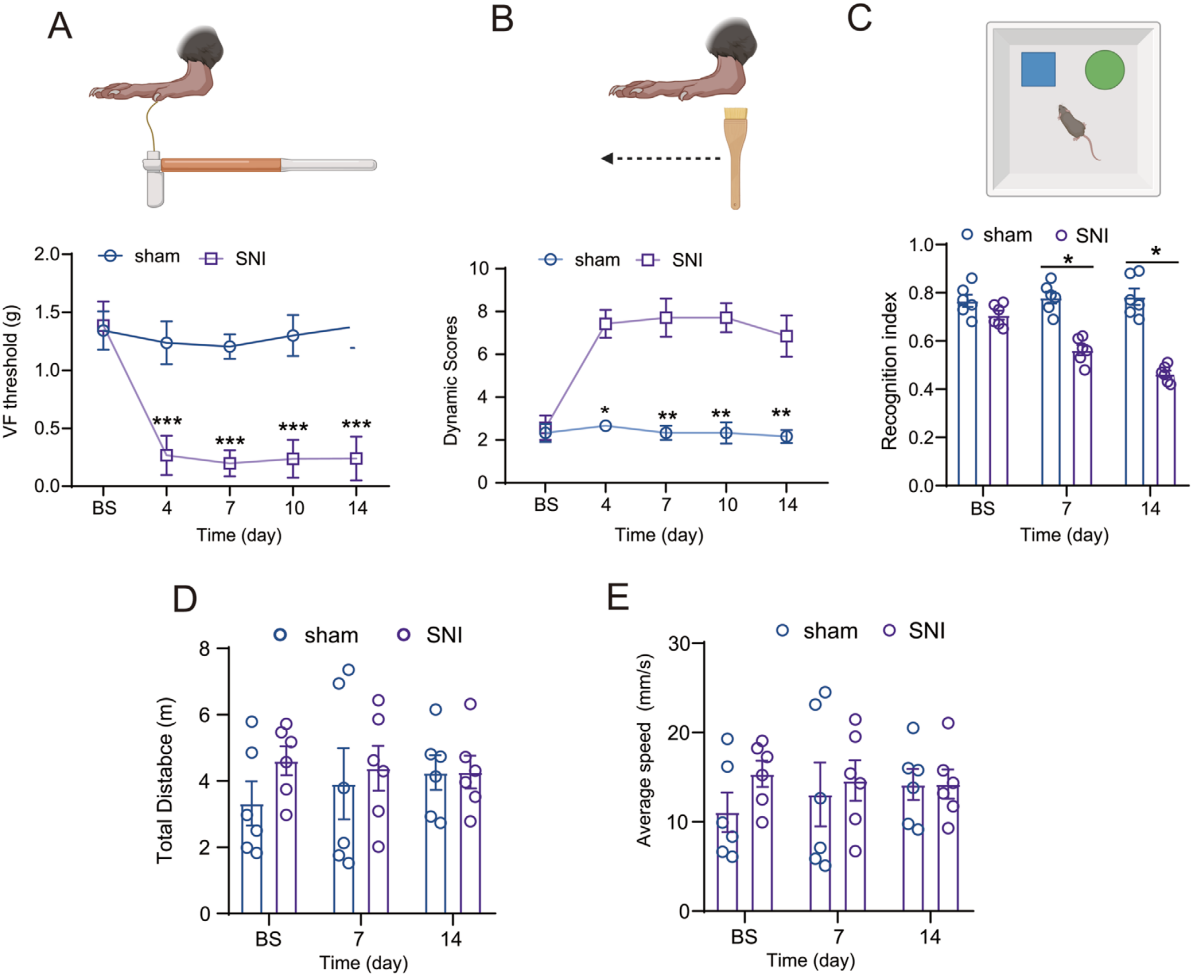
SNI induces nociceptive sensitization and cognitive dysfunction. (A) SNI decreased the tactile threshold in the ipsilateral hind paw to surgery, which was detected at day 4 and continued to day 21 after SNI operation; (SNI, *n* = 8 mice; sham, *n* = 5 mice; Mann–Whitney U test nonparametric test). ***p < 0.001 compared with the sham group. Schematic illustration of Von Frey hair stimulation is shown at the *top*. (B) SNI enhanced responsiveness to dynamic mechanical stimulation. (SNI, *n* = 7 mice; sham, *n* = 6 mice; Mann–Whitney U nonparametric test). *p < 0.05; **p < 0.01 compared with the sham group. Schematic illustration of brush stroke in the hind paw is shown at the *top*. (C) Schematic presentation of the protocol of the novel object recognition test. Habituation, sampling, and acquisition represented three phases. In the sampling section, two objects were placed in the box. In the test section, a novel object replaced one familiar object. Compared to the sham mice, the novel object recognition index, the ratio of time spent exploring the novel object to total exploration time in the spared nerve injury mice, was significantly reduced at day 7 and day 14 (SNI, *n* = 5 mice; sham, *n* = 5 mice; Student's two‐tailed unpaired t‐test, parametric test). *p < 0.05 compared with the sham group.

Behavioral assessments revealed significant mechanical hypersensitivity in SNI mice, as demonstrated by decreased 50% mechanical withdrawal thresholds (Figure 1A, Von Frey test) and enhanced responsiveness to dynamic mechanical stimulation (Figure 1B, brush test). These nociceptive alterations emerged by postoperative day 4 and persisted through day 14 (Figure 1A,B). Concurrent cognitive deficits were observed in novel object recognition testing, with SNI mice showing significantly reduced discrimination indices compared to sham‐operated controls at days 7 and 14 post‐injury (Figure 1C). SNI did not affect the locomotor activity parameters, including total distance traveled (Figure 1D) and average velocity (Figure 1E). This comorbidity of persistent nociceptive sensitization and cognitive impairment was fully established by day 7 and maintained through day 14 post‐SNI.

### 3.2 SNI‐Induced Comorbidity Is Associated With Decreased Neurogenesis and Reduced Granule Cell Excitability in the Dorsal DG

**FIGURE 2:**
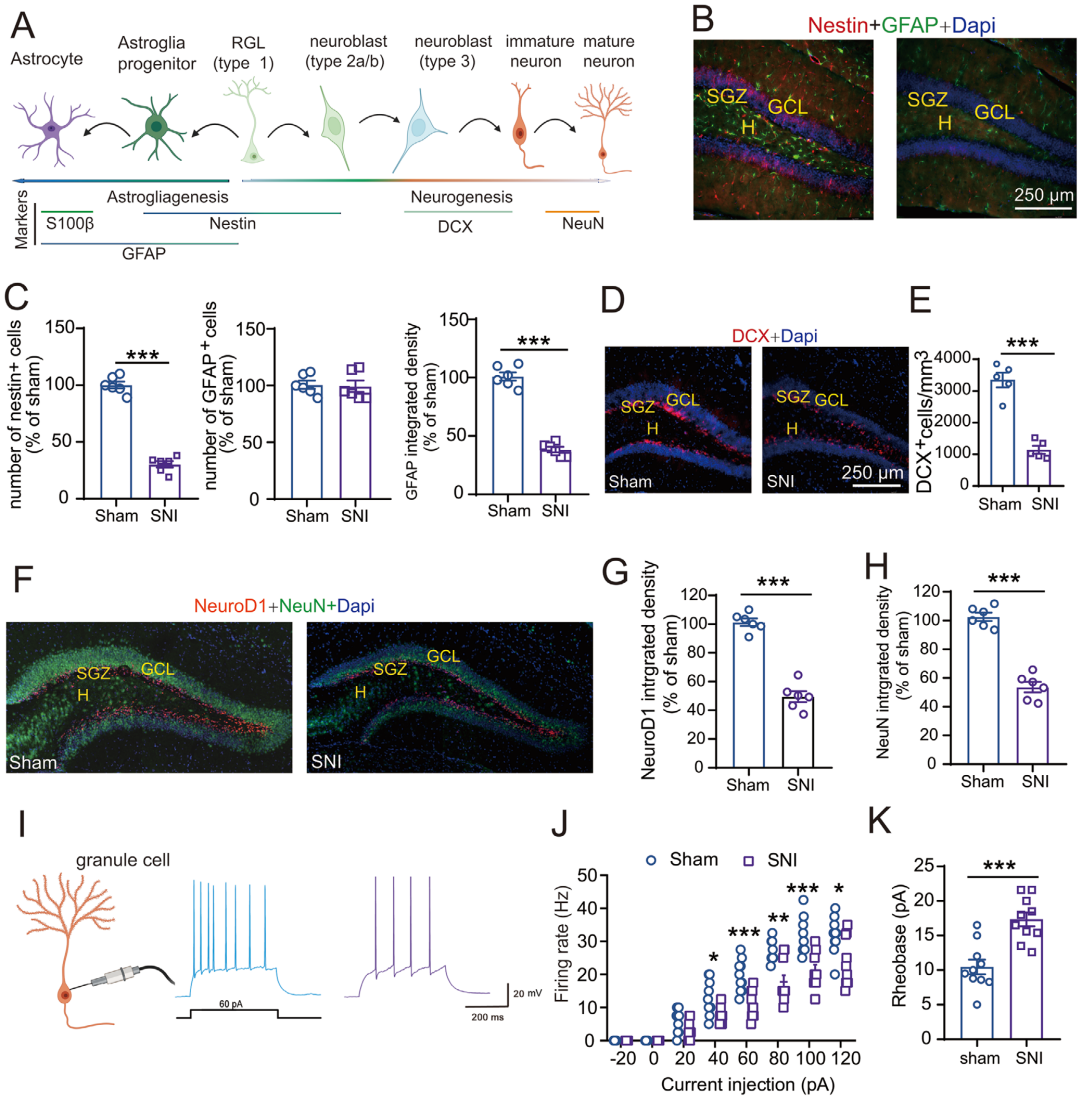
SNI reduced neurogenesis and decreased intrinsic excitability of granule cells in the dorsal DG. (A) Markers of astrogliagenesis and neurogenesis. (B‐C) Immunofluorescence staining showed that expression of the astrocyte markers GFAP was markedly decreased in SNI mice (Figure 2B,C, *n* = 5 mice/group, 3 ~ 4 sections/mouse). Student's two‐tailed unpaired t‐test, ***p < 0.001 compared with the sham group. (D‐E) DCX^+^ immature neuron density was reduced at day 10 after SNI. Student's two‐tailed u, *p* < 0.0001 compared with the sham group. (F‐H) Expression of NeuroD1 and NeuN was decreased at day 10 after SNI. Student's two‐tailed unpaired t‐test, ***p < 0.0001 compared with sham group. (I–J) Examples of the action potential responses to positive current injection recorded in granule cells in dorsal DG from sham (*blue*) and SNI (*purple*) mice (I) and the number of spikes (J) induced by injected currents in the contralateral dorsal DG granule cells from sham (*n* = 10 cells, 5 mice) and SNI mice (*n* = 10 cells, 4 mice; Mann sham *n* = 10 cells). *p < 0.05; **p < 0.01, ***p < 0.001 vs. sham group. (K) The rheobase of dorsal DG granule cells was increased after SNI. Student's two‐tailed unpaired *t‐*test, ***p < 0.001 versus. sham group.

Neural stem cells can differentiate into neurons via neurogenesis or generate glial cells through gliogenesis, with distinct cellular markers expressed at specific developmental stages (Figure 2A). To investigate SNI‐induced perturbations in dorsal DG plasticity, we combined quantitative immunofluorescence analysis of neurogenic markers with functional electrophysiological assessments at day 10 post‐injury. Neurogenic impairment in SNI mice manifested through four key observations: (1) The number of nestin^+^ RGL cells was substantially reduced, whereas the number of GFAP^+^ cells remained intact, even though the optical intensity of GFAP was markedly decreased in SNI mice (Figure 2B,C). (2) DCX^+^ immature neuron density was reduced (Figure 2D,E, *p* < 0.001; 3344 ± 230.7, 1144 ± 125.6 cells/mm^3^ in sham and SNI mice, respectively), reflecting arrested neuronal differentiation. (3) Transcriptional dysregulation: downregulation of the early neuronal marker NeuroD1 expression (*p* < 0.05), suggesting impaired neurogenic programming (Figure 2F,G). (4) Maturation failure: Attenuated NeuN immunoreactivity in post‐mitotic neurons (Figure 2F,H), consistent with delayed functional integration. Whole‐cell patch‐clamp recordings further revealed profound functional deficits in mature granule cells of SNI mice. Input–output relationships demonstrated a rightward shift in neuronal activation thresholds, with injured animals exhibiting significantly fewer action potentials across depolarizing current steps (20–120 pA; *p* < 0.01 vs. controls; Figure 2I,J). This hypofunctional phenotype was corroborated by an elevation in action potential rheobase (*p* < 0.001; Figure 2K), indicating reduced intrinsic excitability.

These multimodal findings demonstrate that peripheral nerve injury disrupts both developmental neurogenesis and functional circuit integration through coordinated mechanisms, including impaired progenitor maintenance, aberrant transcriptional regulation, and compromised maturation.

### 3.3 SNI Increased pS612 IRS1 Expression in Granule Cells and Decreased IGF‐1 Signaling in the Dorsal DG

Having shown that the differentiation of neurons in dorsal DG was impaired in chronic pain/memory impairment comorbid mice, we further explored the reasons for this impairment. Insulin/IGF‐1 resistance, when serine/threonine residues (e.g., Ser307 and Ser612) are aberrantly phosphorylated instead of tyrosine residues, and thus insulin signaling is attenuated, has been reported to affect neuronal differentiation.

**FIGURE 3:**
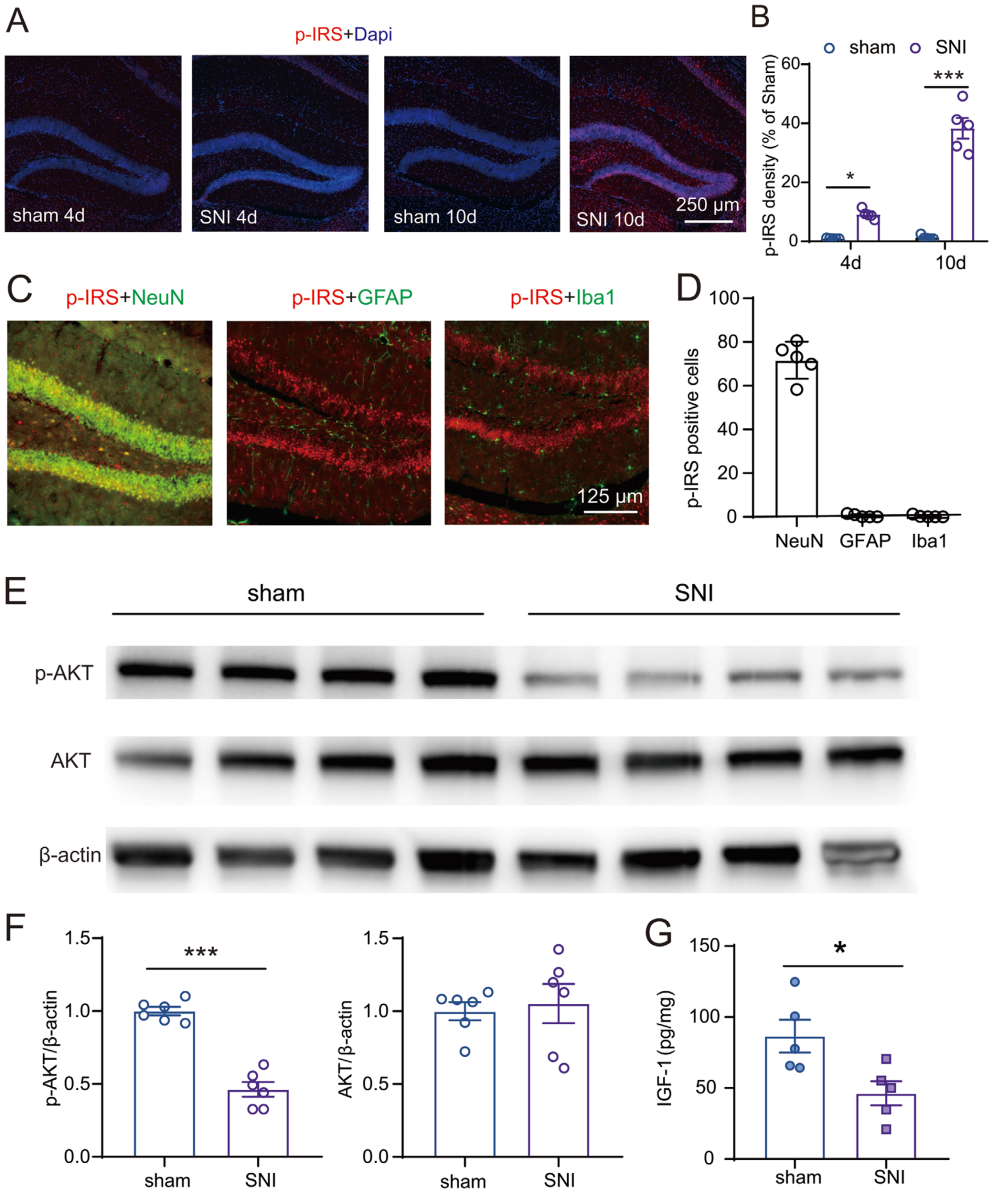
SNI increased pS612 IRS1 expression in granule cells of the dorsal DG. (A) Changes in pS612 IRS1‐IR in the contralateral dorsal DG on days 4 and 10 in sham or SNI mice with comorbidities. (B) Quantification of pS612 IRS1‐IR optic density in the contralateral dorsal DG in sham or SNI mice with comorbidities (*n* = 5/group; one‐way ANOVA followed by post hoc Bonferroni correction, parametric test). (C, D) Percentage of neurons, astrocytes, or microglia in dorsal DG that expressed p‐IRS1 in sham or SNI mice with comorbidities (day 10) (*n* = 5–6/group). E, F AKT phosphorylation was reduced (E) and the level of IGF‐1 (F) was decreased at day 10 after SNI.

To test whether SNI induced IGF‐1 resistance in the dorsal DG, the expression of pS612 IRS1 was detected by immunofluorescence experiments. The results showed that compared to the sham group, the optical density of pS612 IRS1 was significantly upregulated on days 4 and 10 (Figure 3A,B) in SNI comorbidity mice. Double staining further revealed that pS612 IRS1 was increased within NeuN‐positive granule cells of dorsal DG in SNI comorbid mice at day 10, while GFAP‐positive astrocytes and Iba1 positive microglia (Figure 3C,D) remained unaffected. These results suggest that IGF‐1 resistance occurred in neurons following SNI. Furthermore, the phosphorylation pattern at the inhibitory S612 residue of IRS1 was accompanied by diminished AKT phosphorylation (Figure 3E,F) and decreased IGF‐1 expression (Figure 3G) in the dorsal DG at day 10 post‐SNI, indicating impaired downstream insulin/IGF‐1 signaling.

### 3.4 Supplementation of IGF‐1 to Dorsal DG of SNI Mice Alleviated the Pain Sensitization and Cognitive Impairment Induced by SNI, Which Was Abolished by IGF‐1R Antagonist or AKT Inhibitor

As IGF‐1 level was decreased in dorsal DG after the development of nociceptive sensitization and cognitive impairment, we next tested whether exogenous supply of IGF‐1 directly into dorsal DG via a cannula could alleviate the consequences of nociceptive sensitization and memory deficits induced by SNI.

**FIGURE 4:**
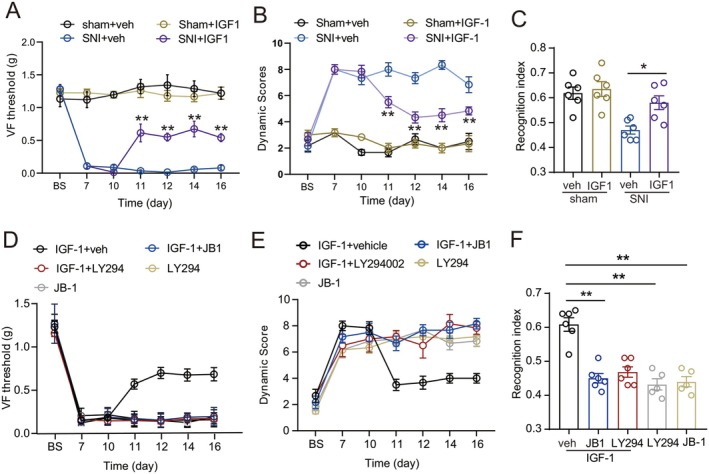
IGF‐1 alleviated the mechanical allodynia and cognitive impairment induced by SNI, which was abolished by JB‐1 or LY294002. (A‐C) Effect of microinjection of IGF‐1 (0.5 μg/μl in 0.3 μL) into the contralateral dorsal DG of SNI mice with comorbidities (day 10) on the tactile threshold in the ipsilateral hind paw, brush stoke evoked dynamic score and novel object recognition index (*n* = 5–6/group, Mann–Whitney U nonparametric test). *p < 0.05, **p < 0.01 compared with the vehicle‐treated SNI group. (D‐F) The therapeutic effects of IGF‐1 on the tactile threshold, dynamic score, and novel object recognition index in SNI mice were blocked by pretreatment with either the IGF‐1R antagonist JB‐1 or the AKT inhibitor LY294002. In contrast, administration of JB‐1 or LY294002 alone did not alter these baseline measures in SNI mice (*n* = 5–6/group, Mann–Whitney U nonparametric test). **p < 0.01 compared with vehicle and IGF‐1‐treated SNI group.

At day 10 after SNI, when the nociceptive sensitization and memory deficit comorbidity had been established, microinjection of IGF‐1 (1 μg/μL, 0.5 μL) into the contralateral dorsal DG in mice (to the injury), on day 10 after SNI, and then daily for 4 days, significantly increased the tactile threshold (Figure 4A) and the dynamic score (Figure 4B) to brush stroke in the ipsilateral hind paw at day 11–16, and on the novel object recognition index (Figure 4C) at day 11 after SNI. In contrast, IGF‐1 administration in sham‐operated mice elicited minimal changes in tactile threshold (Figure 4A), dynamic responses (Figure 4B), or recognition indices (Figure 4C). Critically, pretreatment with the IGF‐1R antagonist JB‐1 (0.8 μM in 0.5 μL) or the AKT inhibitor LY294002 (0.8 μM in 0.5 μL) abolished the therapeutic effects of IGF‐1 in SNI mice (Figure 4D–F), implicating IGF‐1R/AKT signaling in the observed rescue of nociceptive and cognitive deficits. Neither JB‐1 nor LY294002 injected alone affected the tactile threshold, the dynamic score, or the recognition indices (Figure 4D–F).

### 3.5 IGF‐1 Increased the Neurogenic Capacity and the Firing Rate of Granule Cells of SNI Mice

**FIGURE 5:**
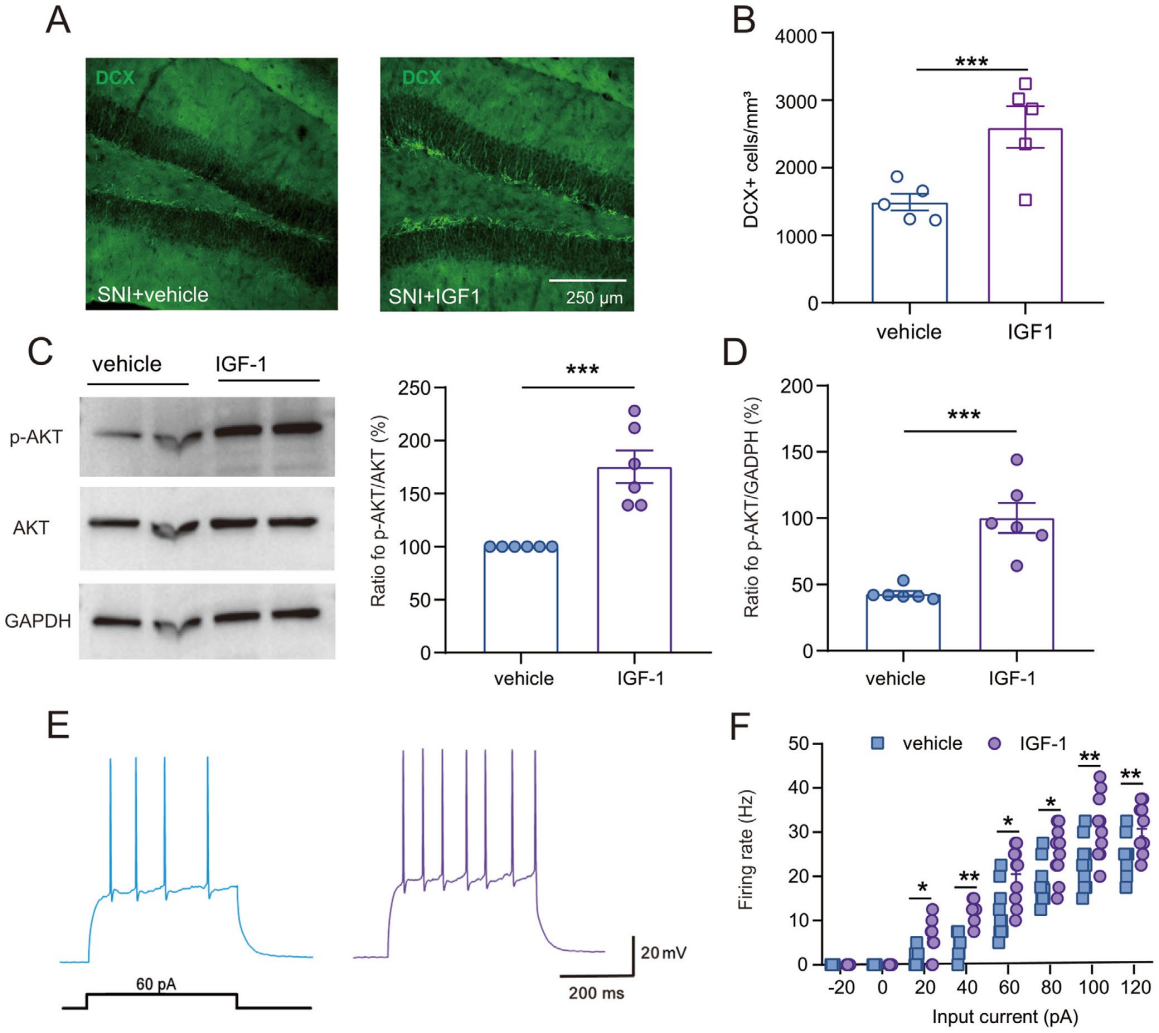
IGF‐1 increased the neurogenic capacity and the firing rate of granule cells of SNI mice. (A‐B) IGF‐1 treatment enhanced the firing rate of granule cells in DG of SNI mice (*n* = 10 cells, 5 mice; Mann–Whitney U test). *p < 0.05, **p < 0.01 compared with the vehicle‐treated SNI group. (C‐D) IGF‐1 treatment enhanced the number of DCX^+^ immature neurons (*n* = 5–6/group, Student's two‐tailed unpaired *t‐*test). ***p < 0.001 compared with the vehicle‐treated SNI group. (E, F) Compared to the vehicle, IGF‐1 increased the expression of p‐AKT but not AKT in mice with comorbidity (one‐way ANOVA followed by post hoc Bonferroni correction, *n* = 6/group). ***p < 0.001 compared with the vehicle‐treated SNI group.

Immunofluorescence staining showed that the number of DCX^+^ immature neurons increased to 2604 ± 307.2/mm^3^ in IGF‐1‐treated SNI mice, compared to 1491 ± 123.2/mm^3^ in vehicle‐treated SNI mice (Figure 5A,B, *p* < 0.001), and the p‐AKT level recovered to 175.3% ± 15.43 of the vehicle‐treated SNI group (Figure 5C,D, *p* < 0.001). Furthermore, electrophysiological recordings revealed that IGF‐1 enhanced the excitability of granule cells in SNI mice (Figure 5E,F). Input–output relationships demonstrated that IGF‐1‐treated mice exhibited significantly more action potentials across depolarizing current steps (20–120 pA; Figure 5F).

These findings indicate that localized IGF‐1 supplementation can transiently reverse SNI‐induced neuropathic pain and cognitive deficits through PI3K/AKT‐dependent restoration of granule cell homeostasis and neurogenesis.

### 3.6 Expression of WNT3 Was Downregulated in Dorsal DG Astrocytes in SNI Comorbid Mice

**FIGURE 6:**
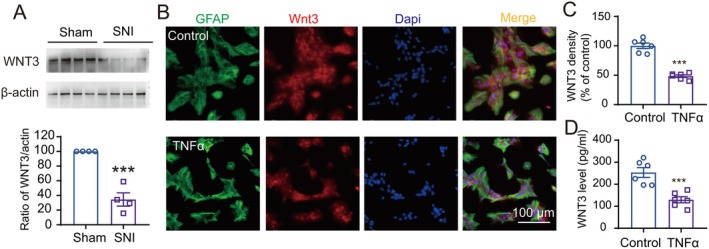
Expression of WNT3 was downregulated in dorsal DG astrocytes in SNI comorbid mice. (A) The bands show the level of WNT3 and β‐Actin in different groups as indicated. The histograms show the ratio of Wnt3 to β‐Actin, which were then compared to the sham group (*n* = 4/group, one‐way ANOVA followed by post hoc Bonferroni's test). ***p < 0.001 compared with the sham group. (B, C) Immunohistochemistry analysis showed that treatment of TNFα but not vehicle for 24 h in cultured astrocyte from dorsal hippocampus decreased the expression of WNT3 (*n* = 5–6/group, Student's two‐tailed unpaired *t‐*test). ***p < 0.001 compared with the vehicle‐treated (control) group. (D) ELISA results showed that secretion of WNT3 was decreased after treatment of TNFα but not vehicle for 24 h (*n* = 6/group, Student's two‐tailed unpaired t‐test). ***p < 0.001 compared with the vehicle‐treated (control) group.

WNT3 secreted from astrocytes can promote NSC differentiation in a paracrine manner; we next investigated whether peripheral nerve injury disrupts the secretion of WNT3. Western blot analysis showed that compared with the sham group, the expression of WNT3 in dorsal DG was decreased at day 10 after SNI (Figure 6A). In cultured astrocyte from dorsal hippocampus, treatment of recombinant murine TNF*α*, a leading proinflammatory cytokine that was released after peripheral nerve injury, could also disrupt the secretion of WNT3, as compared with the vehicle, and the expression of WNT3 in astrocytes was decreased 24 h after 100 ng/mL TNFα application (Figure 6B,C). ELISA further confirmed that the level of WNT3 was downregulated (Figure 6D).

### 3.7 Activation of Astrocytes in Dorsal DG Attenuated Nociceptive Sensitization/Memory Deficits via Increasing WNT3

**FIGURE 7:**
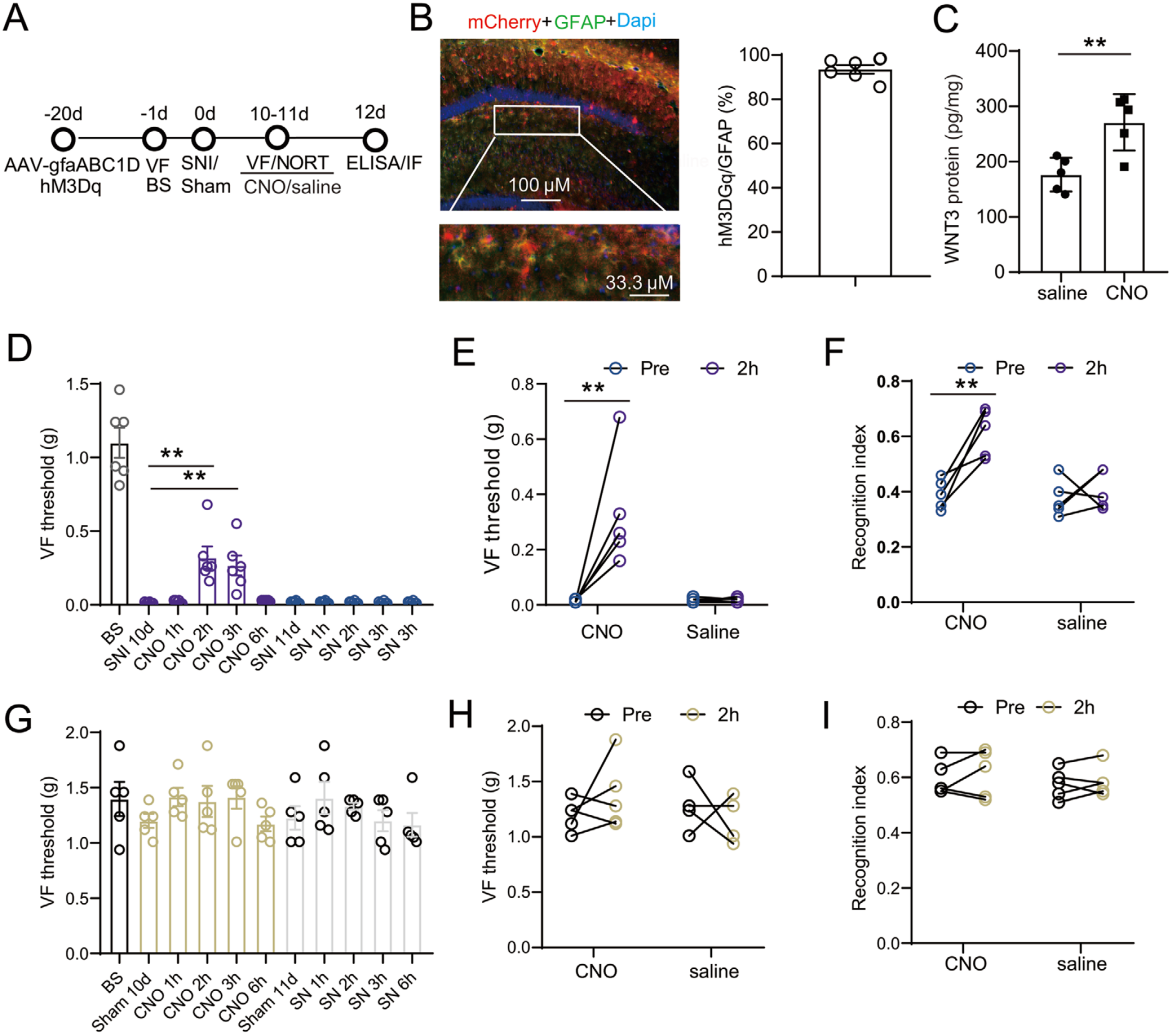
Chemogenetic activation of astrocytes in dorsal DG alleviated nociceptive sensitization and cognitive decline via WNT3. (A) The timeline of the experiments. (B) mCherry‐hM3D(Gq) was localized in n GFAP–marked astrocytes (*n* = 5/group). (C) Effect of intraperitoneal (i.p.) CNO or saline on the level of WNT3 in dorsal DG (*n* = 5/group; Student's two‐tailed unpaired *t*‐test). **p < 0.01 versus saline group. (D‐I) Effect of CNO or saline on the tactile threshold and novel object recognition index in SNI comorbid (D‐F) or sham (G‐I) mice. E and H show the changes in the tactile threshold before and 2 h after treatment of CNO or saline in SNI and sham mice (*n* = 6/group; Friedman nonparametric one‐way ANOVA with Dunn's post‐test). **p < 0.01 at 2 h compared with SNI/sham day 10.

We next examined whether activation of astrocytes in dorsal DG could alleviate the comorbidity of pain and cognitive impairment after SNI. AAV encoding hM3D(Gq) fused to mCherry under the control of the GFAP promoter (pAAV‐gfaABC1D‐hM3D(Gq)‐P2A‐mCherry‐WPRE) was injected into the contralateral dorsal DG of mice. CNO was injected i.p. (1 mg/kg) 21 days later to allow activation of hM3D(Gq) (Figure 7A). Immunofluorescence staining showed that a total of 93.5% ± 1.98% of GFAP‐positive astrocytes in dorsal DG were transfected with the virus (Figure 7B). ELISA showed that compared to saline, i.p. injection of CNO significantly enhanced the secretion of WNT3 (Figure 7C). Behavioral tests showed that 2 h after CNO but not saline injection, the tactile threshold of comorbidity of mice was significantly enhanced at day 10 after SNI surgery (Figure 7D,E). CNO but not saline also enhanced the recognition index in SNI comorbidity mice (Figure 7F). Neither CNO nor saline had any effect on the tactile threshold and the recognition index of sham mice (Figure 7G–I).

### 3.8 Chronic Inhibition of Astrocytes in Dorsal DG Mimicked SNI‐Induced Nociception and Cognitive Deficits and Disruption of Neurogenesis

**FIGURE 8:**
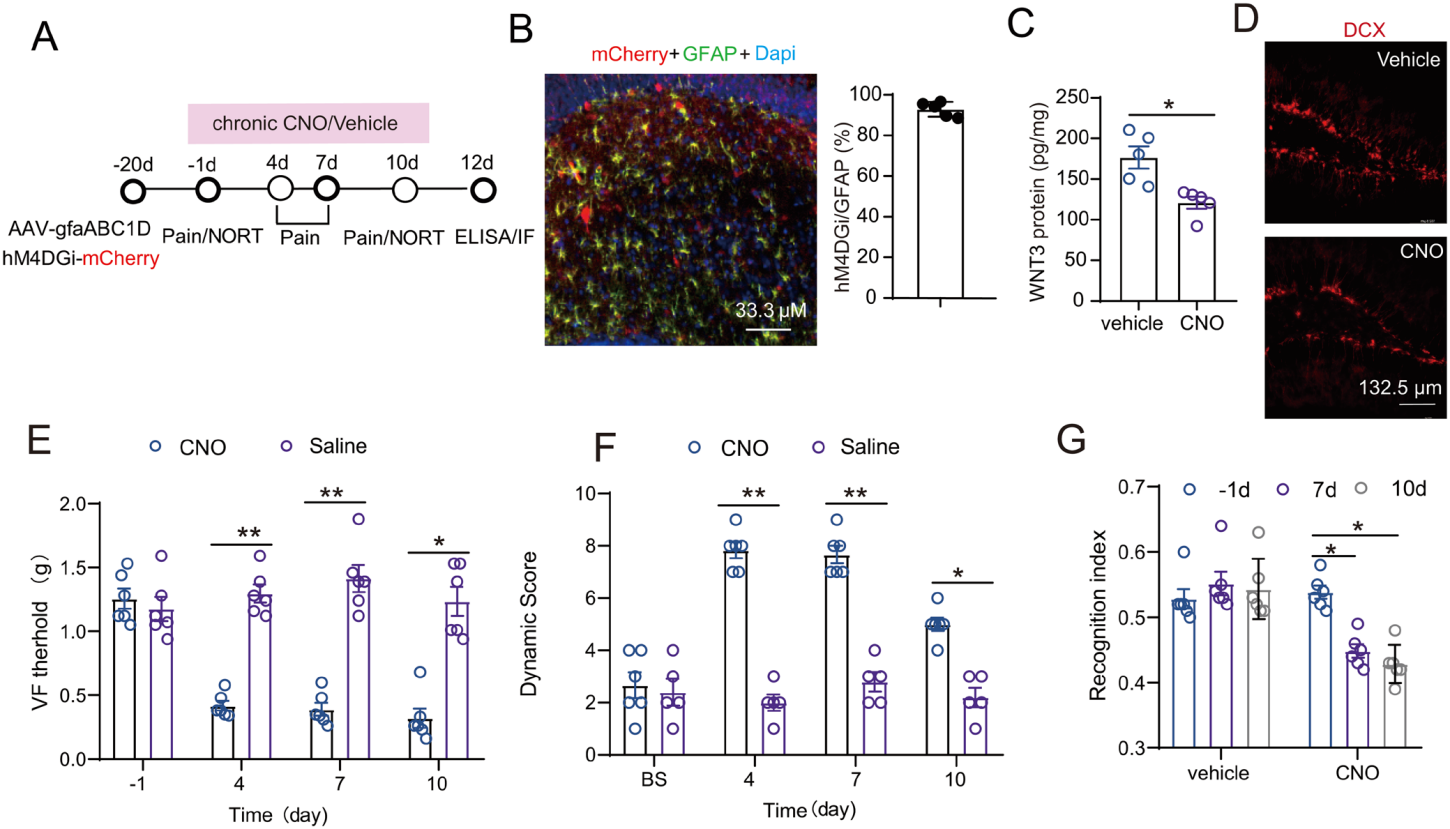
Chemogenetic inhibition of astrocytes in dorsal DG alleviated nociceptive sensitization and cognitive decline via WNT3. (A) The timeline of the experiments. (B) mCherry‐hM4D(Gi) was localized in GFAP–marked astrocytes (*n* = 5/group). (C‐D) Effect of chronic CNO or vehicle treatment for 7 days on the level of WNT3 or DCX^+^ immature neurons in dorsal DG (*n* = 5/group; Student's two‐tailed unpaired *t*‐test). *p < 0.05 versus saline group. (E‐G) Effect of chronic CNO or vehicle treatment for 7 days on the tactile threshold, the dynamic score and the novel object recognition index (*n* = 6/group; Friedman nonparametric one‐way ANOVA with Dunn's post‐test). **p < 0.01 at 2 h compared with SNI/sham day −1.

We next examined whether chronic inhibition of astrocytes in dorsal DG could induce pain sensitization and cognitive impairment, similar to SNI. AAV encoding hM4D(Gi) fused to mCherry under the control of the GFAP promoter (pAAV‐gfaABC1D‐hM4D(Gi)‐P2A‐mCherry‐WPRE) was injected into the contralateral dorsal DG of mice (Figure 8A). CNO was administered ad libitum in 2% sucrose water at 300 mg/L for 7 days. Immunofluorescence staining showed that a total of 92.9% ± 1.62% of GFAP‐positive astrocytes in dorsal DG were transfected with the virus (Figure 8B). ELISA showed that compared to the vehicle, administration of CNO significantly decreased the level of WNT3 (Figure 8C) and DCX^+^ immature neurons (Figure 8D). Behavioral tests showed that CNO but not the vehicle treatment decreased the tactile threshold and enhanced the dynamic score of mice at days 4, 7, and 10 (Figure 8E,F). The recognition index at days 7 and 10 was also decreased by CNO but not the vehicle treatment (Figure 8G).

### 3.9 Intrahippocampal Administration of WNT3a Into Dorsal DG Alleviated Pain Hypersensitivity and Cognitive Impairments After SNI by Activating AKT

**FIGURE 9:**
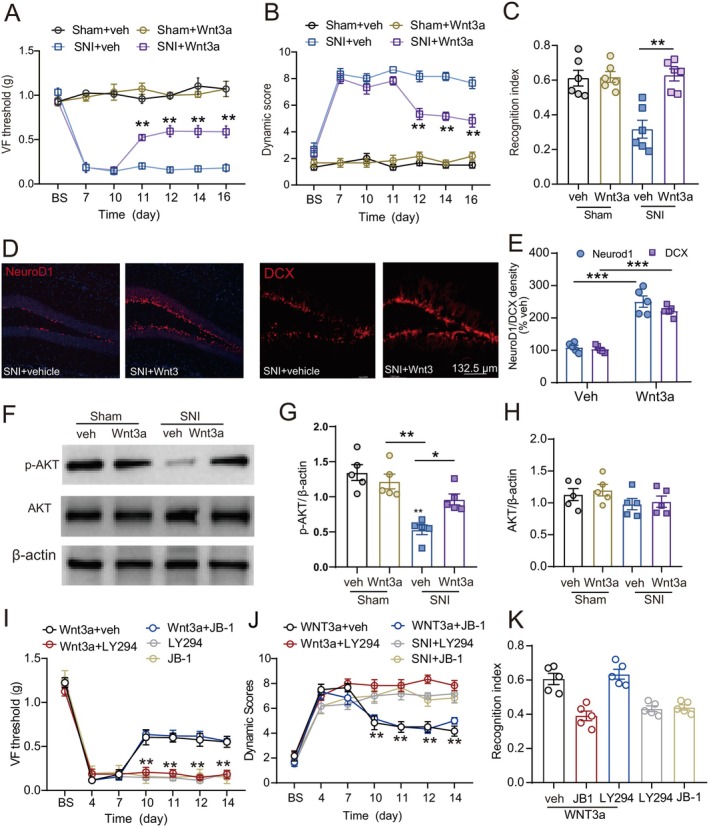
Intrahippocampal administration of WNT3a into dorsal DG alleviated pain hypersensitivity and cognitive impairments after SNI by activating the AKT pathway. (A‐C) The effect of intrahippocampal administration of recombinant WNT3a (200 ng/μl in 0.3 μL, on day 10 after SNI or sham operation, and then daily for 4 days) in the contralateral dorsal DG on 50% paw withdrawal threshold, dynamic score and novel object recognition index (*n* = 5–6/group, Mann–Whitney nonparametric test). ***p < 0.001 compared with the vehicle‐treated SNI group. (D, E) WNT3a treatment in SNI mice enhanced the expression of NeuroD1 and DCX. (F‐H) Western blotting showed that WNT3a treatment in SNI mice enhanced the expression of p‐AKT, but not total AKT. (I‐K) LY294002 but not JB‐1 completely abolished the effects of WNT3a on 50% paw withdrawal threshold, dynamic score and novel object recognition index. Mann–Whitney nonparametric test, **p < 0.01 compared with WNT3a and vehicle‐treated group.

Having found that the level of WNT3 was downregulated after SNI, we next observed whether supplement of WNT3 ligand to the dorsal DG affected the pain/cognition. Intrahippocampal administration of recombinant WNT3a, a canonical WNT ligand (200 ng/μl in 0.3 μL) in the contralateral dorsal DG of nerve‐injured mice, on day 10 after SNI, and then daily for 4 days, significantly enhanced sensory processing and cognitive function, as demonstrated by increased tactile thresholds (Figure 9A), improved dynamic brush stroke response scores in the ipsilateral hind paw (Figure 9B), and elevated novel object recognition indices (Figure 9C) at days 11–16 after SNI. Sham‐operated mice showed minimal responses to WNT3a treatment across all measured parameters (Figure 9A–C). Furthermore, the WNT3a treatment in SNI mice enhanced the expression of NeuroD1 (Figure 9D), DCX (Figure 9E), and p‐AKT (Figure 9F–H), indicating that WNT3a had neurogenic effects. Pharmacological interventions revealed that the PI3K‐AKT inhibitor LY294002 (0.8 μM in 0.5 μL) but not IGFR1 antagonist JB‐1 (0.8 μM in 0.5 μL) completely abolished WNT3a‐mediated improvements in neuropathic pain behaviors and cognitive impairment in SNI mice (Figure 9I–K). Neither JB‐1 nor LY294002injected alone affected the tactile threshold, the dynamic score, or the recognition indices. These results suggest that WNT3a might indirectly affect downstream effects of IGFR1 by enhancing PI3K/AKT pathway.

## 4 Discussion

Neuropathic pain and comorbid cognitive impairment impose substantial disability—up to 60% of NP patients report memory deficits that worsen quality of life, even when taking commonly prescribed analgesics (e.g., gabapentin, duloxetine); critically, these drugs rarely address the shared neural substrates underlying both phenotypes [34]. These deficits are likely driven by pain‐related maladaptive plasticity in the dorsal DG, a well‐recognized node linking pain processing and memory regulation [2, 3]. Prior work has established two key observations. First, chronic pain directly impairs dorsal DG neurogenesis [5, 6], a process essential for memory function; second, insulin‐like growth factor‐1 (IGF‐1) and WNT signaling pathways are core regulators of adult dorsal DG neurogenesis [10, 11, 15]. However, most prior studies have investigated these pathways or the dorsal DG's role in isolation—focusing on either pain or cognition, not their comorbidity—leaving unresolved how peripheral nerve injury coordinates IGF‐1/WNT signaling to drive dual pathology. Our study fills this void by identifying a novel astrocyte‐mediated WNT3/IGF‐1 signaling axis in the dorsal DG as a critical mechanistic link between peripheral nerve injury and the concurrent development of nociceptive sensitization and memory impairment.

### 4.1 A Convergence of Neurogenic and Signaling Deficits Contributes to Peripheral Nerve Injury‐Induced Comorbidity

Our behavioral findings confirmed that SNI produced persistent mechanical hypersensitivity and concurrent cognitive impairment, consistent with clinical observations of trigeminal neuralgia patients exhibiting hippocampal volume loss and cognitive deficits [4]. This comorbidity is correlated with robust disruptions of neurogenesis in dorsal DG. Specifically, we observed a decrease in Nestin^+^ radial glia‐like (RGL) neural stem cells, a reduction in DCX^+^ immature neurons, downregulated NeuroD1 expression, and impaired integration of NeuN^+^ mature neurons. These deficits align with prior reports that chronic pain suppresses hippocampal neurogenesis [3, 6] and extend this knowledge by demonstrating that such impairments specifically affect the dorsal DG, a subregion critical for spatial memory and pain modulation [2, 35]. Notably, while the total number of astrocytes remained intact following SNI, individual astrocytes exhibited reduced GFAP expression. This suggests that SNI does not cause astrocyte loss, but rather induces a transition to a hypoactive state, potentially compromising their structural support and neuroprotective functions due to impaired GFAP synthesis. The depletion of the primary RGL stem cell pool provides a pivotal, upstream mechanism that directly compromises the entire neurogenic cascade, logically accounting for the subsequent reductions in neuronal progenitors and mature neurons.

Although we did not directly assess the potential contribution of increased newborn neuron apoptosis, the discovery of a diminished stem cell reservoir offers a strong and parsimonious explanation for the global impairment of neurogenesis.

Electrophysiological recordings further revealed that SNI reduces intrinsic excitability of dorsal DG granule cells, as evidenced by elevated rheobase and decreased action potential firing. This functional deficit likely contributes to both cognitive impairment (via disrupted memory circuit integration) and nociceptive sensitization through impaired descending pain modulation pathways [22]. The coordinate disruption of neurogenesis and granule cell excitability suggests a fundamental breakdown in dorsal DG circuit homeostasis following peripheral nerve injury—consistent with reports of synaptic integrity loss and dendritic spine abnormalities in other pain models [2].

### 4.2 IGF‐1 Resistance in Dorsal DG Granule Cells as a Key Driver of Comorbidity

A critical insight from our study is the identification of IGF‐1 resistance in dorsal DG granule cells as a central event in SNI pathogenesis. We demonstrate that SNI induces aberrant serine phosphorylation of IRS1 at S612—a modification associated with insulin/IGF‐1 signaling impairment [14] —specifically in NeuN^+^ neurons, concurrent with reduced IGF‐1 expression and AKT hypophosphorylation. This neuron‐specific IGF‐1 resistance provides a mechanistic explanation for the observed neurogenic deficits, as IGF‐1 signaling is well established to promote neuronal differentiation, synaptic plasticity, and survival [11, 12].

The functional significance of this pathway is underscored by our rescue experiments: local administration of IGF‐1 into the dorsal DG reversed both nociceptive hypersensitivity and cognitive impairment in SNI mice, with these effects abrogated by IGF‐1R antagonism or PI3K/AKT inhibition. This confirmed that IGF‐1 acted through canonical IGF‐1R/PI3K/AKT signaling to restore neurogenesis (increased DCX^+^ cells) and granule cell excitability—directly linking signaling restoration to functional circuit recovery. These findings extended prior work on IGF‐1's neuroprotective roles [36, 37] by demonstrating its capacity to reverse established pain–memory comorbidity when delivered to the dorsal DG, highlighting a potential therapeutic target.

### 4.3 Astrocyte‐Derived WNT3 Is a Paracrine Regulator of the Pain–Memory Axis

Our study further identified astrocyte‐secreted WNT3 as a critical upstream regulator of dorsal DG homeostasis in the context of nerve injury. We showed that SNI reduced WNT3 expression in dorsal DG astrocytes, an effect recapitulated by TNF‐α exposure in cultured astrocytes—consistent with reports that peripheral injury triggers central neuroinflammation and astrocyte dysfunction [19, 20, 38]. Activation of dorsal DG astrocytes via chemogenetic stimulation (hM3DGq) increased WNT3 secretion and alleviated SNI‐induced comorbidity, while inhibition of dorsal DG astrocytes via hM4DGi reduced WNT3 secretion and mimicked SNI‐induced comorbidity, directly linking astrocyte function to WNT3‐mediated neuroprotection.

Mechanistically, exogenous WNT3a administration mimicked the therapeutic effects of IGF‐1, restoring nociceptive thresholds, cognitive function, and neurogenesis in SNI mice. Notably, the effects of WNT3a are abolished by PI3K/AKT inhibition but not IGF‐1R blockade, indicating that WNT3 potentiates PI3K/AKT activation independently of IGF‐1R. This aligns with prior reports of crosstalk between WNT and IGF‐1 pathways [16, 17]. Our findings thus identified astrocyte WNT3 as a paracrine regulator of neurogenesis and granule cell function, with its deficiency contributing to SNI‐induced comorbidity.

Our previous work has shown that TNF‐*α* was increased significantly in the plasma, CSF, and hippocampus [20, 39]. Nerve injury also activates the sympathetic nervous system, enhances the activity of lipolytic enzymes, and leads to increased release of free fatty acids (FFA) from adipose tissue [4]. Thus, after peripheral nerve injury, increased TNF‐α/FFAs activate JNK‐1 and mTOR pathways, directly promoting IRS‐1 phosphorylation at inhibitory serine sites (Ser307/Ser612/Ser616). Such aberrant phosphorylation disrupts insulin/IGF‐1 signaling by blocking interactions between IRS‐1 and insulin/IGF‐1 receptors, leading to PI3K/AKT pathway suppression. Inhibition of the PI3K/AKT pathway then suppresses synthesis of synaptic protein [21] and ultimately impairs synaptic plasticity.

### 4.4 The WNT3/IGF‐1 Axis as a Unifying Mechanism for Pain–Memory Comorbidity

Integrating these observations, we propose a model where peripheral nerve injury induces a “double hit” to dorsal DG homeostasis: (1) inflammatory signals (e.g., TNF‐*α*) suppress astrocytic WNT3 secretion, reducing paracrine support for neurogenesis; (2) concurrent IGF‐1 resistance in granule cells (via IRS1 S612 phosphorylation) impairs neuronal responsiveness to remaining trophic signals. Together, this dual impairment disrupts neurogenic progression, reduces granule cell excitability, and drives the development of pain–memory comorbidity.

This model explains why restoration of either pathway—via IGF‐1 supplementation or astrocyte activation/WNT3a administration—partially reverses the pathology: IGF‐1 bypasses the resistance mechanism to activate PI3K/AKT, while WNT3a enhances downstream signaling independently of IGF‐1R. In neural systems, this interplay acquires spatiotemporal specificity. IGF‐1 triggers sustained PI3K‐AKT pathway activation in hippocampal neurons via IGF‐1R signaling [40]. Notably, astrocyte‐derived WNT3a extends the temporal window for IGF‐1R activation, suggesting synergistic regulation. However, peripheral nerve injury disrupts this coordination through dual mechanisms: (1) marked downregulation of IGF‐1 expression and (2) impaired astrocytic WNT secretion. The concurrent loss of both ligands may abolish IGF‐1R signaling efficacy, highlighting the interdependence of WNT and IGF‐1 in maintaining neural metabolic homeostasis. Based on the previous studies and our results that IGF‐1 increased neurogenesis and excitability of mature granule cells, as well the effects of WNT3a were abolished by AKT inhibitor, we propose that peripheral nerve injury disrupts this astrocytic signaling axis through dual mechanisms: suppressing WNT3 secretion while inducing IGF‐1 resistance. Therapeutic synergy of these pathways warrants exploration, as combined targeting may yield more durable effects.

#### 4.4.1 Our Findings Have Significant Translational Potential

The dorsal DG‐specific nature of these deficits suggests that targeted delivery strategies (e.g., intranasal or hippocampal‐directed) for IGF‐1 or WNT3 agonists could avoid systemic side effects. Additionally, identifying upstream regulators of astrocyte WNT3 secretion (e.g., anti‐TNF‐α therapies) or IRS1 phosphorylation (e.g., serine phosphatase activators) may provide alternative therapeutic avenues.

While local dorsal DG interventions show promise, translational challenges demand caution. First, IGF‐1's blood–brain barrier (BBB) penetration is modulated by serum‐binding proteins (IGFBP and others), and intracranial pumps are clinically limited to severe conditions (e.g., refractory epilepsy) due to infection risks. Novel strategies—such as IGF‐1 fusion with BBB‐transporting peptides (e.g., transferrin receptor ligands [41])—are needed to enable non‐invasive delivery. Second, WNT3a has a short half‐life, requiring frequent dosing. Long‐acting formulations (e.g., lipid‐encapsulated WNT3a or heparin‐bound agonists) could sustain dorsal DG levels.

### 4.5 Limitations and Future Direction

First, while we demonstrate IRS1 phosphorylation in neurons, the precise upstream kinases mediating this modification (e.g., JNK, IKK*β*), remains unknown. Second, long‐term effects of IGF‐1/WNT3a administration require evaluation, as chronic neurogenesis modulation may have unintended consequences. Third, we exclusively used male mice, but clinical NP shows marked sex differences—female mice have estrogen‐dependent IGF‐1R expression and WNT3 sensitivity. Validating the WNT3/IGF‐1 axis in female models is critical to avoid gender bias in translation. Finally, we used DCX to assess neurogenesis but did not measure dorsal DG granule cell death (e.g., TUNEL staining for apoptosis). If SNI induces neuronal loss, it could exacerbate dysfunction independently of reduced neurogenesis—future studies must distinguish these two processes.

In conclusion, this study identifies a novel astrocyte–neuron signaling axis in the dorsal DG that couples peripheral nerve injury to pain–memory comorbidity. By targeting the WNT3/IGF‐1/AKT pathway, we provide proof of concept for a mechanistically informed therapeutic strategy to address both the sensory and cognitive components of neuropathic pain.

## Author Contributions

Xuhong Wei and Yuxin Qiu conceived the study, designed the experiments, and wrote manuscripts. Yajie An, Ying Wu, Xiangyong Li, Taihe Zhou, Heming Liu, and Yao Xiao performed most of the experiments and analyzed the data. Wenyu Lai assisted with the experiments. All authors read and approved the final manuscript.

## Funding

This study was supported by the National Natural Science Foundation of China (81870969 to X.W.), Natural Science Foundation of Guangdong Province of China (2022A1515011989 and 2025A1515011088 to X.W., 2022A1515012597 to X.L.) and Guangdong Provincial Key Laboratory of Brain Function and Disease (2023B1212060018 to Y.Q.).

## Ethics Statement

All experiments and procedures involving animals were approved according to guidelines established by IACUC, Sun Yat‐sen University (Approval Nos: SYSU‐IACUC‐2020‐B0884).

## Consent

The authors have nothing to report.

## Conflicts of Interest

The authors declare no conflicts of interest.

## Supporting information


**Figure S1:** Supporting Information.

## Data Availability

The data that support the findings of this study are available on request from the corresponding author. The data are not publicly available due to privacy or ethical restrictions.
