# Supplementary material for: Impaired WNT3/IGF‐1 Signaling in Dorsal Dentate Gyrus Contributes to Chronic Pain‐Related Cognitive Impairment
Source: CNS Neurosci Ther. 2025 Dec 18;31(12):e70714. doi: 10.1002/cns.70714 (PMC12715354; doi:10.1002/cns.70714)
Supplement: Supplementary file 1 — Figure S1: Supporting Information. [file CNS-31-e70714-s001.pdf]

Full unedited gel for Figure 3E

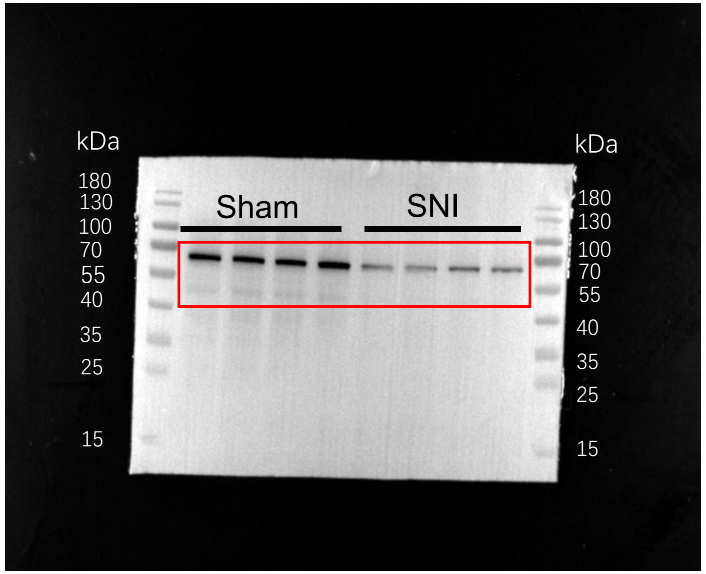

p-AKT

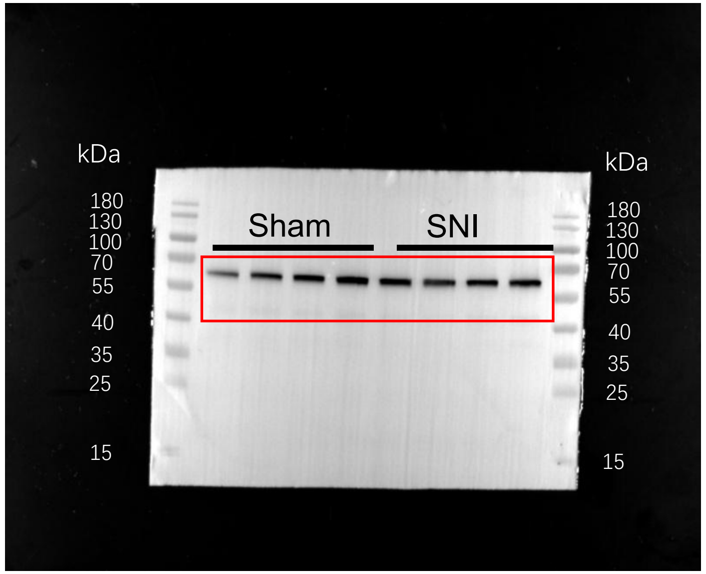

AKT

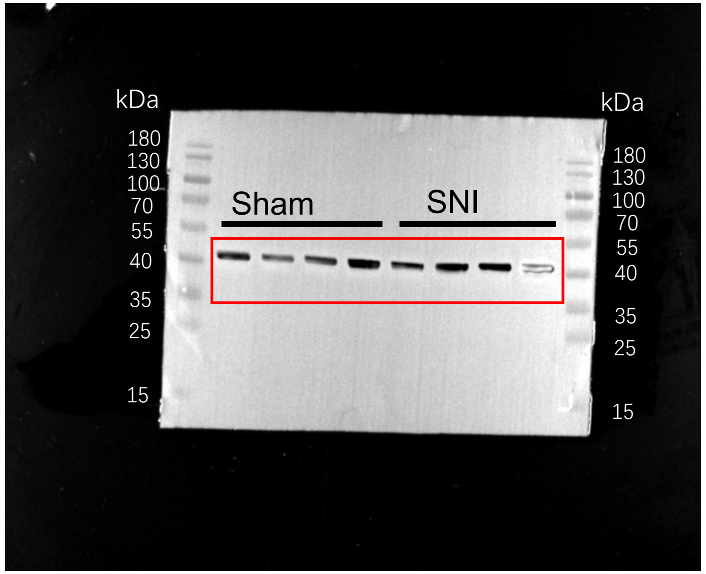

β-actin

Full unedited gel for Figure 5C

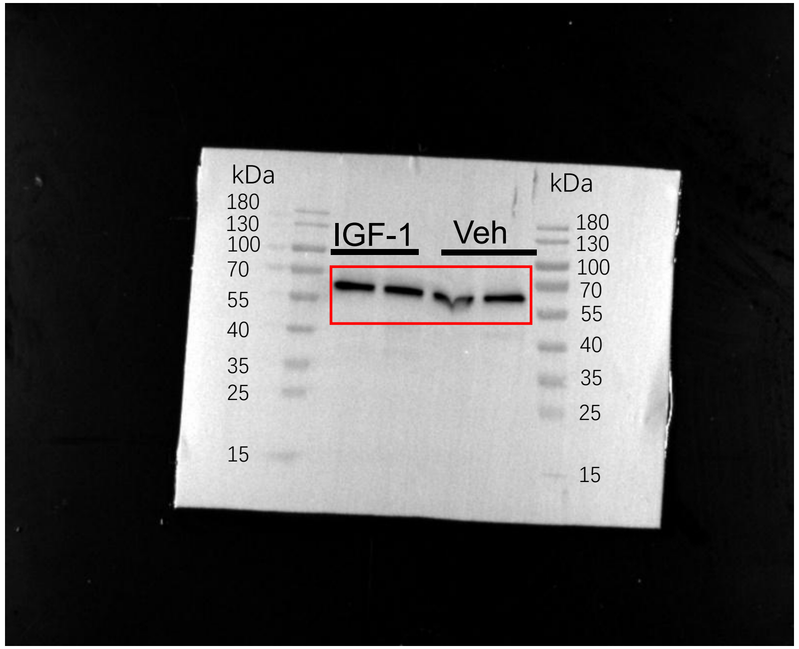

AKT

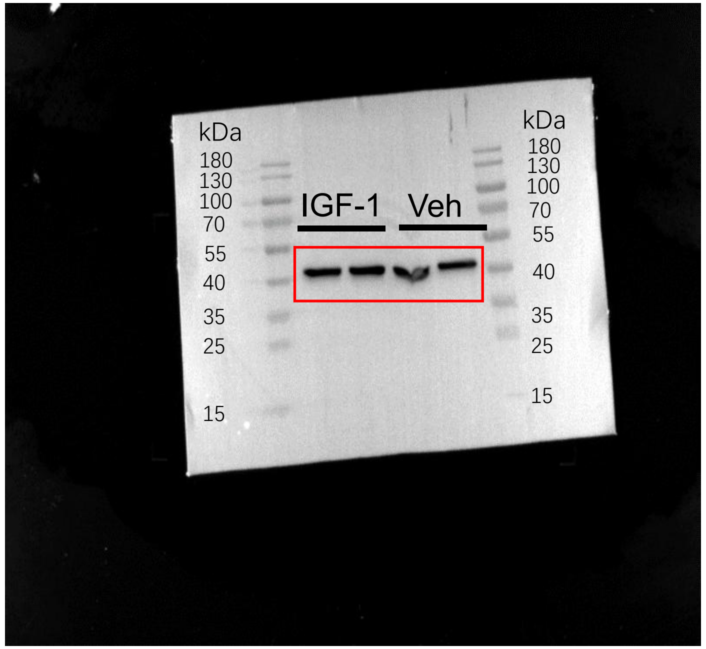

β-actin

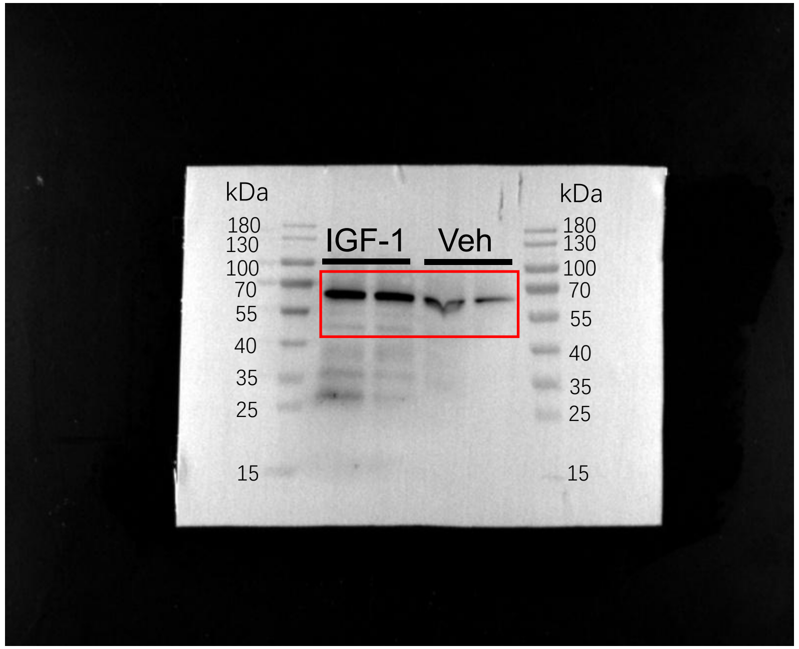

p-AKT

Full unedited gel for Figure 6A

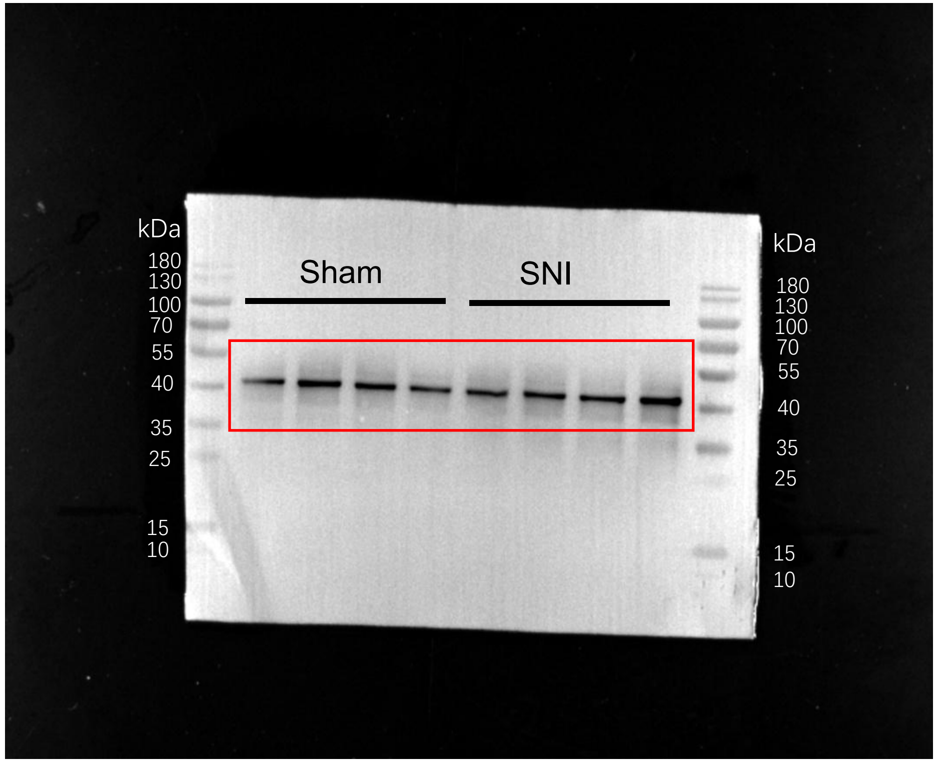

β-actin

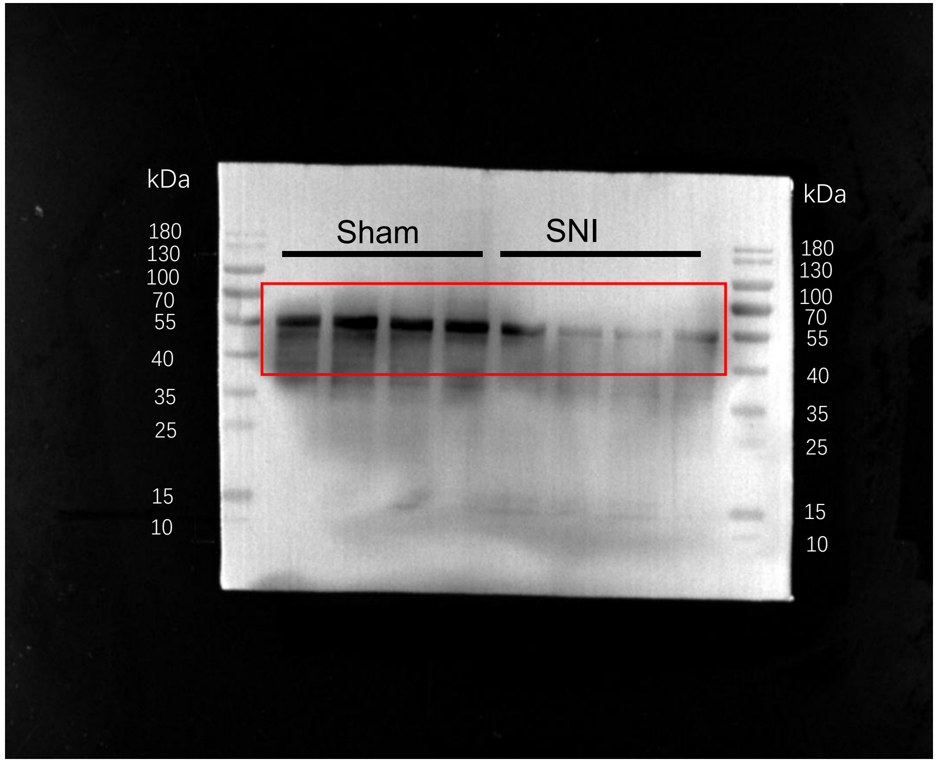

WNT3

Full unedited gel for Figure.9F

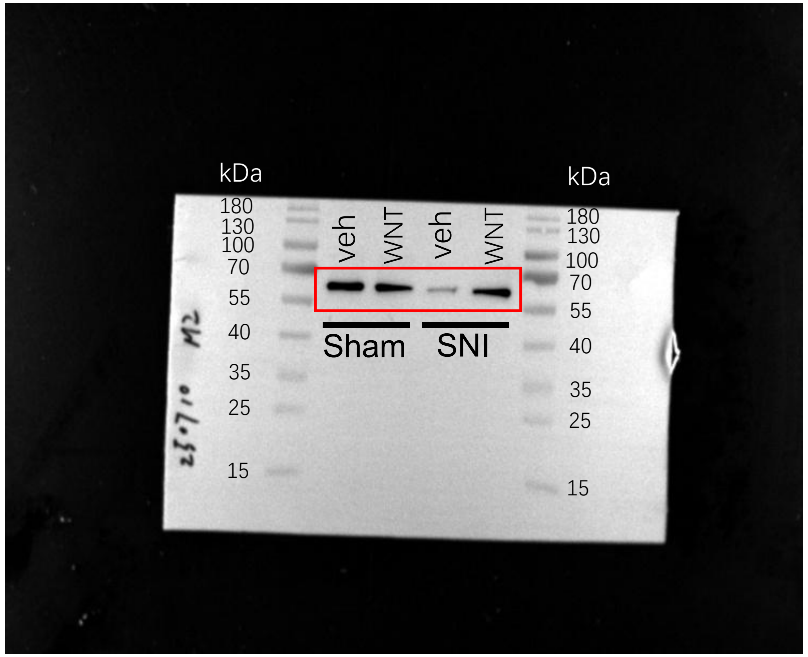

p-AKT

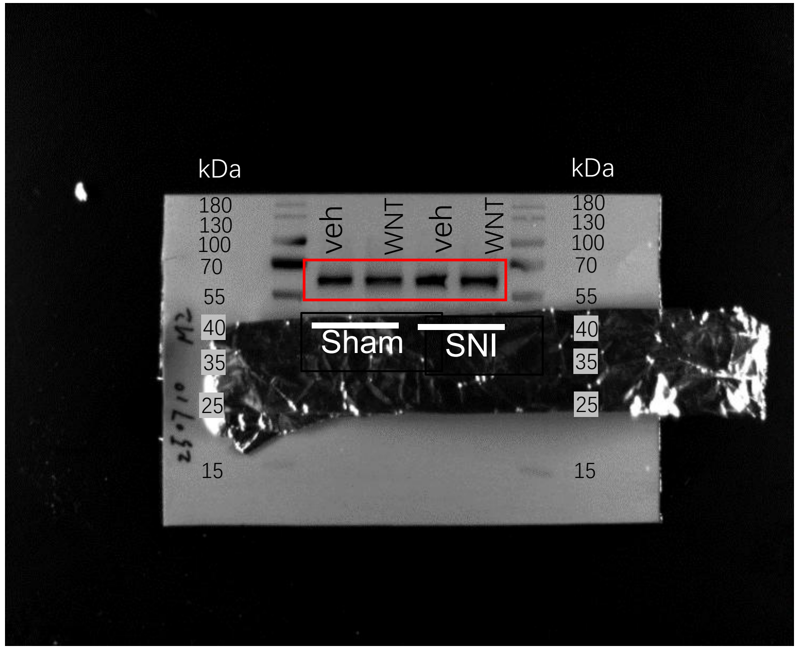

AKT

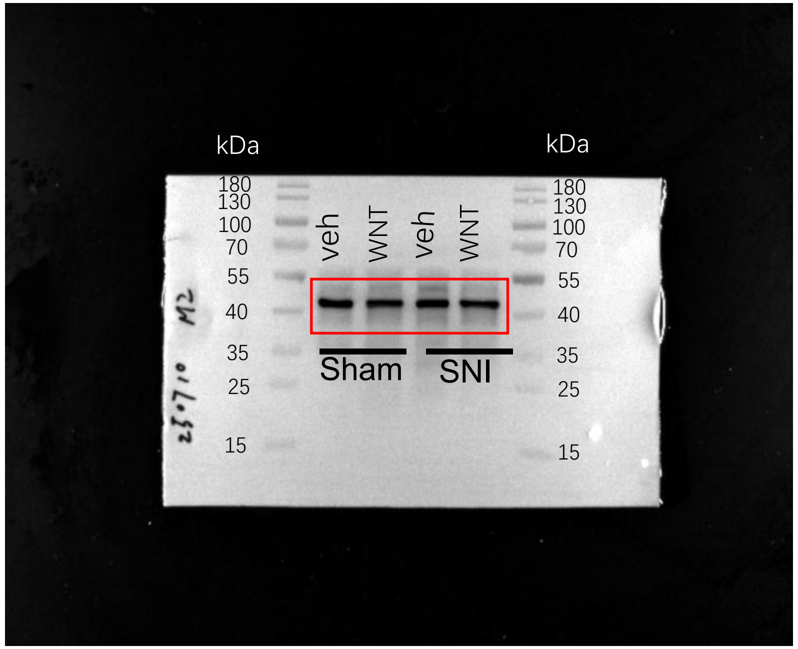

β-actin
